# ASO Author Reflections: Predictors of Pelvic Lymphocele Formation After Lateral Pelvic Node Dissection for Rectal Cancer

**DOI:** 10.1245/s10434-024-16459-3

**Published:** 2024-11-04

**Authors:** Joseph Mathew, Avanish Saklani

**Affiliations:** 1 Department of GI Surgical Oncology and Minimal Access Surgery, HealthCare Global Enterprises Ltd (HCG), Bangalore, India; 2 Division of Colorectal Oncology, Department of Surgical Oncology, Tata Memorial Centre, Mumbai, India

In locally advanced rectal cancers associated with clinically significant lateral pelvic lymphadenopathy, chemoradiotherapy alone often fails to achieve adequate local disease control^1^ necessitating selective lateral pelvic node dissection (LPLND) with total mesorectal excision (TME).^2^ However, this procedure, especially after neoadjuvant radiotherapy, can be technically challenging due to the complex anatomy of the pelvic sidewall and the vascular variations encountered.^3^

Among the complications associated with LPLND, pelvic lymphoceles are regarded the most common, and while predominantly subclinical, these collections can potentially lead to significant morbidity as a result of secondary infection or pressure effects on adjacent organs.^4^ Although anticipating these risks and enhancing post-procedural surveillance can benefit these patients, identifying those at a higher predisposition for lymphocele development at the outset remains crucial.

In the study cohort of 183 patients with lateral node-positive rectal cancer who underwent TME with LPLND between 2014 and 2023, 18% experienced clinically significant morbidity. Pelvic lymphoceles, the most prevalent complication across the postoperative period with a peak incidence within 30 days of surgery, were detected in 21.3% of patients overall, with 7.1% being symptomatic and requiring intervention. On logistic regression performed to identify the potential risk factors for lymphocele formation, the multivariate model comprising body mass index greater than 25 kg/m^2^, receipt of neoadjuvant radiotherapy, open surgical approach, and greater nodal harvest was found to be statistically significant.^5^

Nevertheless, most lymphoceles tend to resolve spontaneously over time, and statistical models that predict the likelihood of complications arising in asymptomatic collections would have greater clinical utility in facilitating the timely implementation of therapeutic interventions. Prospective studies with predefined radiologic screening protocols would determine the precise incidence of these collections and the margin of benefit offered by active surveillance with its associated costs. This in turn would promote the formulation of effective screening strategies and facilitate the individualization of patient follow-up.
